# Characterization data of palladium-alumina on activated biochar catalyst for hydrogenolysis reactions

**DOI:** 10.1016/j.dib.2021.107591

**Published:** 2021-11-19

**Authors:** Lakshmiprasad Gurrala, M. Midhun Kumar, Changyub Paek, R. Vinu

**Affiliations:** a Department of Chemical Engineering and National Center for Combustion Research and Development, Indian Institute of Technology Madras, Chennai 600036, India; b Corporate Strategic Research, ExxonMobil Research and Engineering, Annandale, NJ 08801, United States

**Keywords:** Biochar, Catalyst, Palladium, Instrumental analysis

## Abstract

This article presents experimental data on the techniques used for the characterization of Pd-Al_2_O_3_ supported on activated biochar (2Pd-5Al/ABC) catalyst. The reported data is collected as a part of the research on the 2Pd-5Al/ABC catalyst used for lignin hydrogenolysis [1]. The data on X-ray powder diffraction, ammonia-temperature programmed desorption, pyridine diffuse reflectance infrared Fourier transform spectroscopy, and high-resolution scanning electron microscopy of various catalysts are valuable to study the changes in surface morphology and acidity upon metal loading. The data from thermogravimetric analysis, X-ray photoelectron spectroscopy, and scanning electron microscopy-energy dispersive X-ray spectroscopy are also provided to understand the thermal stability, ionic state of various metals and elemental composition of the catalyst, respectively. The data provided can be used for developing novel catalysts from renewable biochar, and the characterization of noble metal-metal oxide loaded catalysts can aid researchers to design composite catalytic materials for various applications.

## Specifications Table

**Table:** 

Subject	Chemical Engineering: Catalysis
Specific subject area	Development of catalyst for green transformation of waste feedstock's to valuable chemicals
Type of data	Figures
How the data were acquired	XRD (Bruker D8 Discover), TGA (SDT Q600, T.A. Instruments), NH_3_–TPD (Micromeritics Autochem-2920), Pyridine diffuse reflectance infrared Fourier transform spectroscopy (DRIFT, Thermofisher Scientific NICOLET iS50 FTIR spectrometer), XPS (PHI5000 Version Probe III (ULVAC-PHI)), HRSEM (Hitachi S-4800) and HRTEM (JEM-2100 Plus, JEOL, Japan). Raw profiles and images were collected for the biochar, activated biochar and metal loaded activated biochar.
Data format	Raw and processed data
Description of data collection	X-ray diffraction profiles were collected in 2θ range of 10–90°, TGA in N_2_ ambience at a heating rate of 5 °C min^−1^, acidity of the catalysts was measured using NH_3_–TPD up to 800 °C using a thermal conductivity detector (TCD). Pyridine-DRIFT spectrum was recorded at 240 °C. EDS images were obtained using HRTEM JEM-2100 Plus instrument operated at 200 kV.
Data source location	Indian Institute of Technology Madras, Chennai, India.
Data accessibility	Repository name: Mendeley Data Data identification number (doi): http://dx.doi.org/10.17632/v38nbb9rtp.3 Direct URL to data: http://dx.doi.org/10.17632/v38nbb9rtp.3
Related research article	Authors’ names: L. Gurrala, M. M. Kumar, A. Yerrayya, P. Kandasamy, P. Castaño, T. Raja, G. Pilloni, C. Paek, R. Vinu Title: Unraveling the reaction mechanism of selective C9 monomeric phenols formation from lignin using Pd-Al_2_O_3_-activated biochar catalyst Journal: Bioresource Technology, http://dx.doi.org/10.1016/j.biortech.2021.126204

## Value of the Data

• The biochar obtained from biomass pyrolysis has a significant potential to be used as a catalyst support. Furthermore, the development of composite catalysts with Lewis acidic metal oxide supported on renewable carbon is vital for the production of chemicals and fuel molecules via hydrogenolysis and hydrodeoxygenation.
• The detailed characterization methodology of biochar-derived catalyst (Pd-Al/ABC) is essential for researchers working in the field of green chemistry, renewable energy, and catalysis for the production of fine chemicals.
• The present data can be used to gain fundamental insights on the properties of metal loaded activated biochar catalyst. The structural understanding can be used to probe the elementary reactions occurring on the catalyst active sites at different reaction conditions, and to develop structure-activity relationships.

## 1 Data Description

**Fig. 1:**
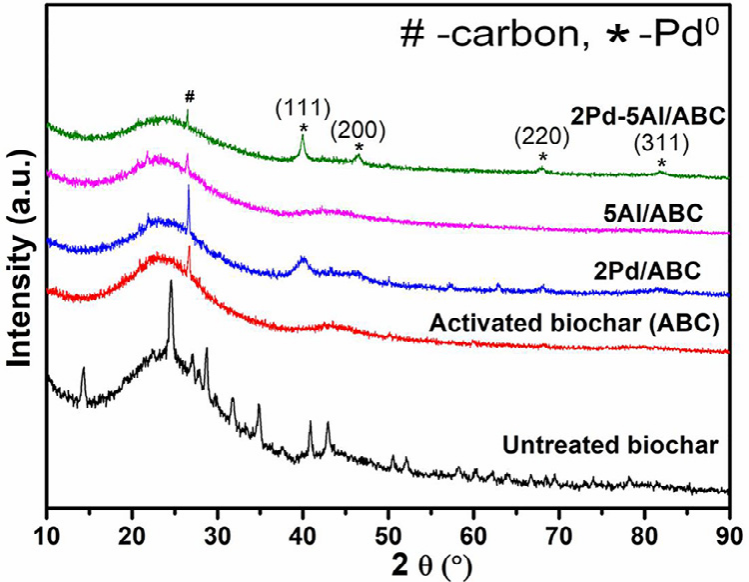
XRD profiles of untreated biochar, ABC, 2Pd/ABC, 5Al/ABC and 2Pd-5Al/ABC catalysts.

**Fig. 2:**
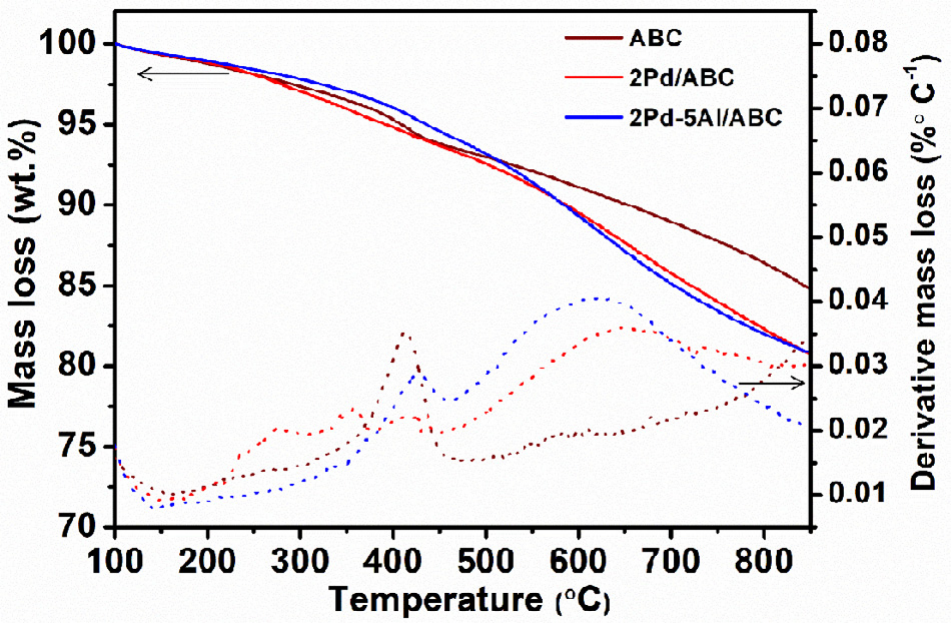
TG mass loss and differential mass loss profiles of ABC and Pd-loaded catalysts in presence of nitrogen.

**Fig. 3:**
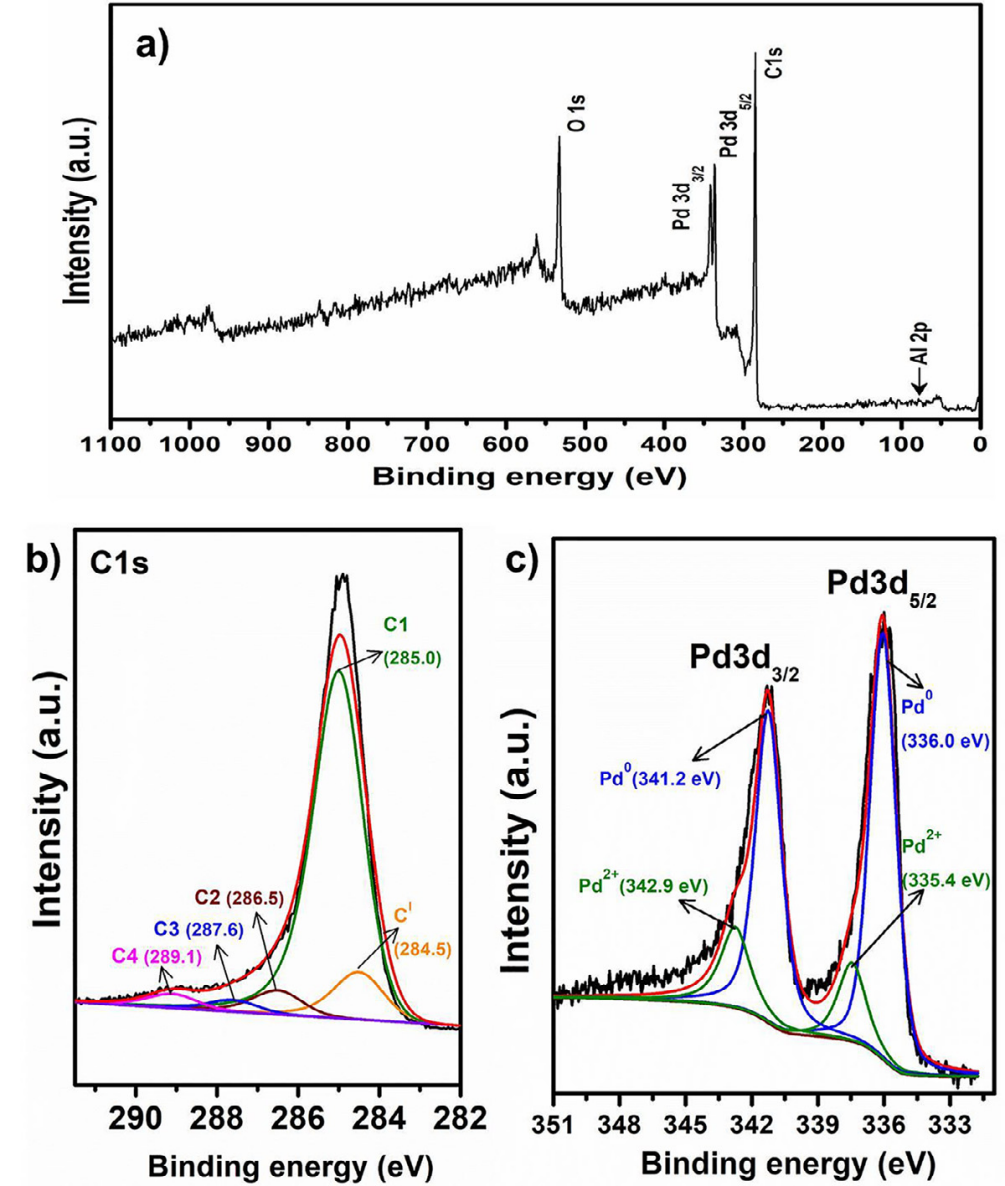
XPS profiles of 2Pd-5Al/ABC catalyst. (a) Survey spectrum, (b) C1s, and (c) Pd3d.

The chemically activated biochar (ABC) was prepared from biochar, which was obtained as a by-product from co-pyrolysis of biomass and waste plastic [2], [3], [4]. Al and Pd metals were loaded on ABC sequentially using wetness impregnation method [5]. The synthesized catalyst was analyzed to gain morphological and structural information. The raw and processed data of 2θ vs intensity values are reported in Mendeley data [6]. Fig. 1 shows the powder XRD profiles of the untreated biochar, ABC and metal loaded ABC catalysts. The XRD profile of untreated biochar shows crystalline diffractions due to the inorganic constituents in it, while after chemical activation most of the diffractions are absent for ABC. Only one sharp crystalline diffraction was observed at 2θ 26.6°. For Pd loaded catalyst, diffraction at 2θ 39.9, 46.4, 68.0 and 81.8° were observed. The TG mass loss and differential mass loss profiles of ABC and Pd loaded ABC in presence of nitrogen are shown in Fig. 2, and the associated data is presented in Mendeley data [6]. The major mass loss regimes were observed at 300-450 °C and 500-700 °C in the derivative mass loss profiles of ABC, 2Pd/ABC and 2Pd-Al/ABC. The mass loss at 850 °C follows the trend: 2Pd/ABC (19.2%) ∼ 2Pd-Al/ABC (19.1%) > ABC (15.2%). Fig. 3a shows the XPS survey spectrum of reduced 2Pd-5Al/ABC catalyst. The carbon 1s peak at 284.5 eV (C^I^) was taken as the reference [7]. The deconvoluted C1s spectrum shows different carbons corresponding to binding energies (BE) ∼285.0, ∼286.5, ∼287.6 and ∼289.1 eV, and these are labelled as C1, C2, C3 and C4, respectively. The relative area % of each of these deconvoluted peaks corresponding to different carbons are C^I^ (10.8%), C1 (78.1%), C2 (5.4%), C3 (2.6%), C4 (3.1%). Similarly, the XPS of Pd3d was further deconvoluted to 5/2 and 3/2 of Pd^0^ and Pd^2+^, respectively. The BE values for Pd3d are 341.2, 342.9, 335.4, and 336.0 eV. The XPS data of binding energy vs intensity for the survey spectrum, C 1s and Pd 3d are available in Mendeley data [6].

**Fig. 4:**
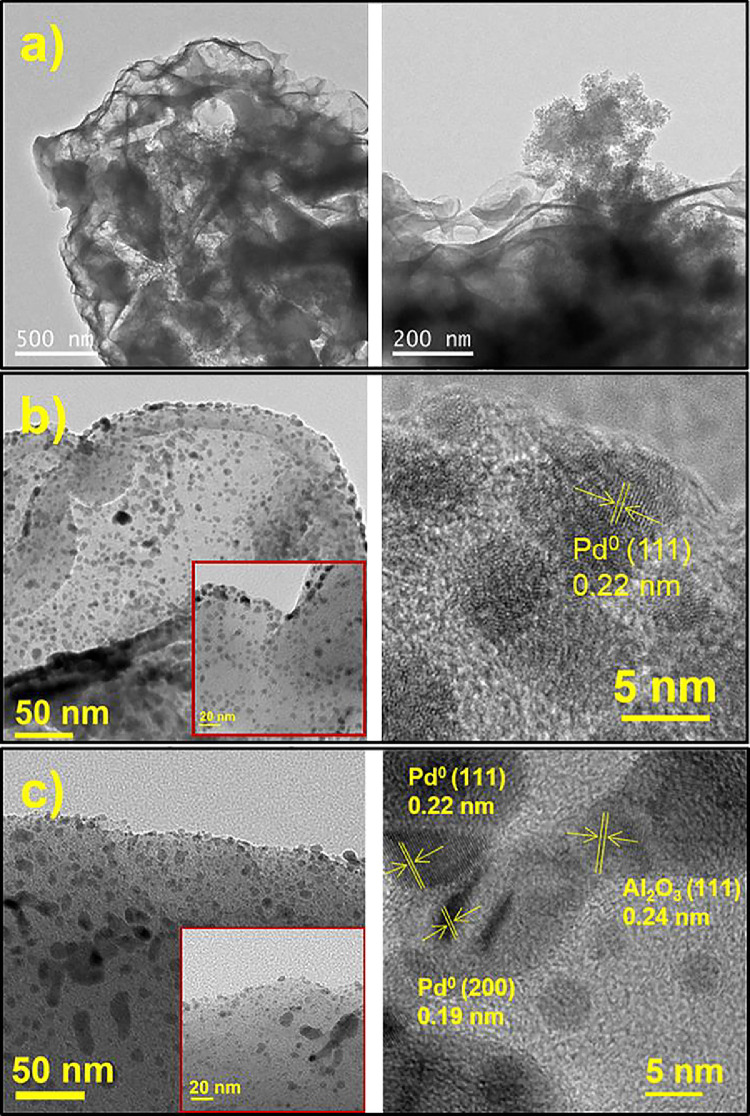
HRTEM images of (a) ABC, (b) Pd/ABC modified from [5], and (c) 2Pd-5Al/ABC catalysts.

**Fig. 5:**
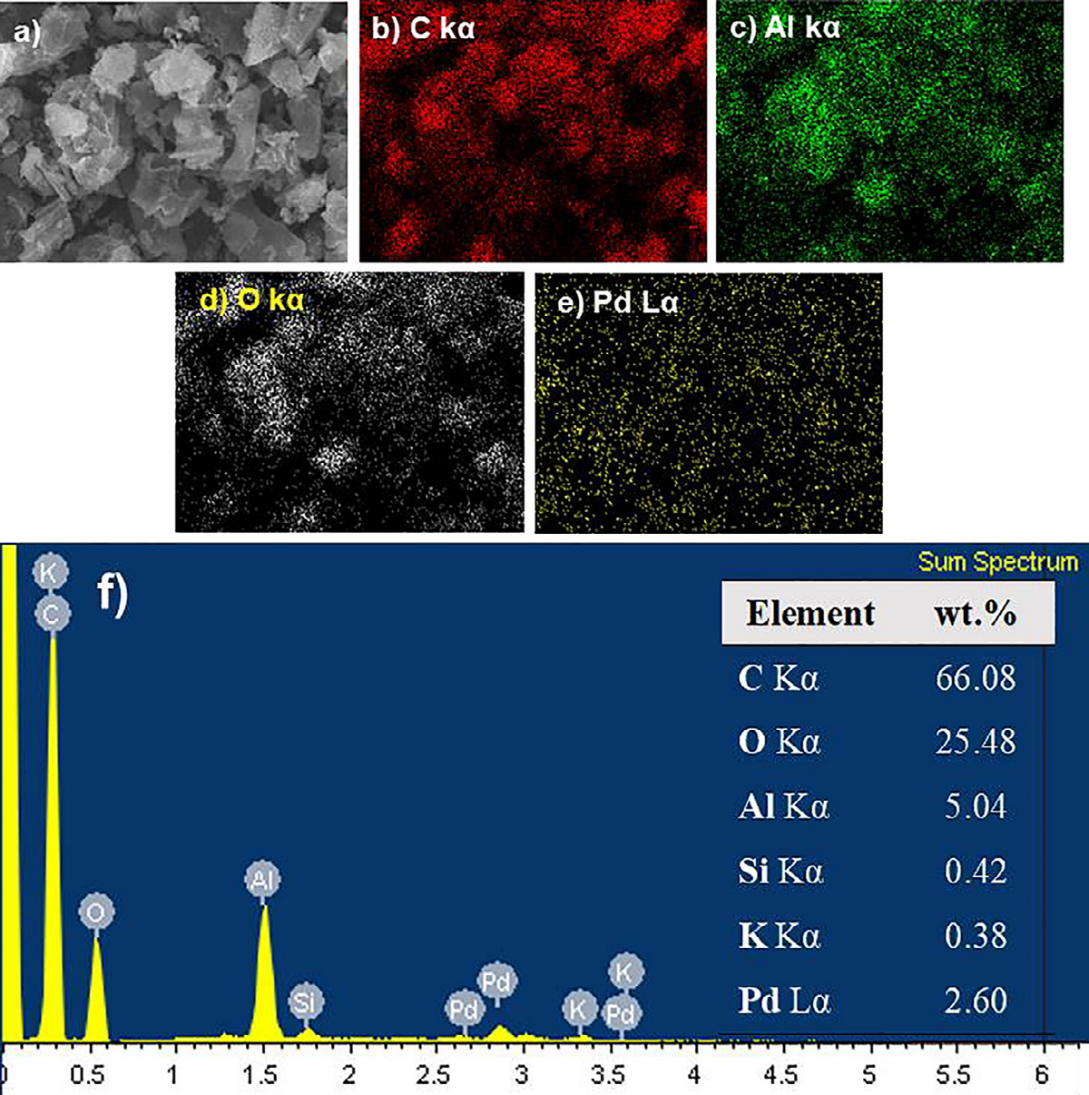
STEM image of (a) 2Pd-5Al/ABC. Elemental mapping images of (b) C, (c) Al, (d) O, and (e) Pd. (f) EDS spectrum of (a) and elemental composition.

**Fig. 6:**
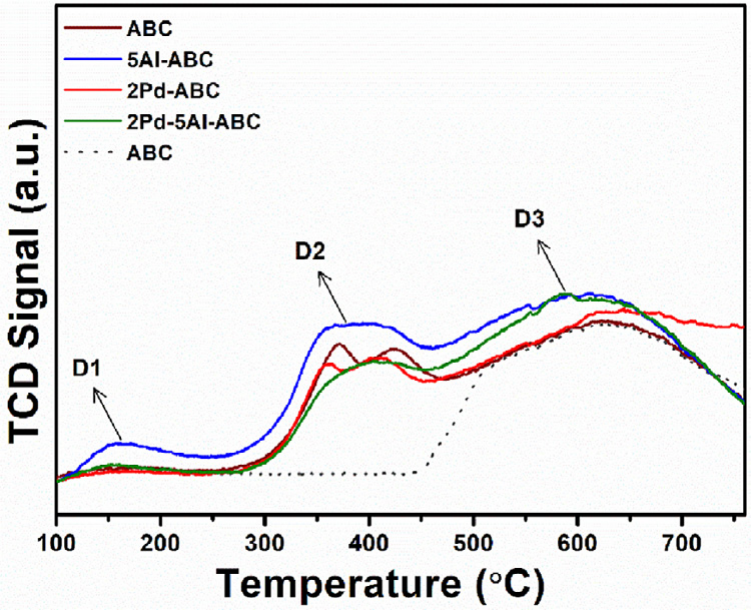
NH_3_-TPD profiles of the catalysts.

**Fig. 7:**
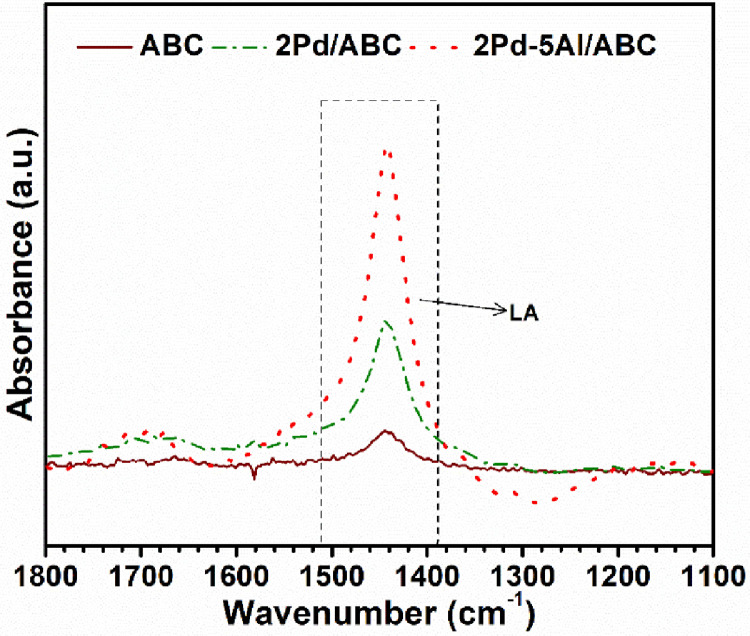
Pyridine-FTIR profiles of the Pd-loaded catalysts (LA: Lewis acidity).

Fig. 4 shows the HRTEM images of the ABC, Pd/ABC, and 2Pd-5Al/ABC catalysts at different magnification. The lattice fringes corresponding to Pd and Al_2_O_3_ are also displayed. Fig. 5 depicts the STEM image of 2Pd-5Al/ABC and elemental mapping images of C, Al, O, and Pd. The EDS spectrum shows the elemental composition of the loaded Al and Pd metals, which is ∼5% and ∼2%, respectively. Fig. 6 shows the TCD signal of NH_3_ evolved from temperature programed desorption from ABC and metal loaded ABC. The actual data of temperature vs TCD signal for the different catalysts are presented in Mendeley Data [6]. Three desorption peaks in the range of 100–250 °C (D1), 250-500/550 °C (D2) and >500/550 °C (D3) are observed. TCD signal of ABC support without adsorbing NH_3_ (dashed line) using NH_3_-TPD shows desorption peak above 500 °C. The pyridine-DRIFT analysis of ABC and Pd-loaded catalysts are shown in Fig. 7. A broad peak was observed at 1440 cm^−1^ in all pyridine-DRIFT profiles. The processed FTIR data of wavenumber vs transmittance are given in Mendeley data [6].

## 2 Experimental Methods

A Bruker D8 Discover diffractometer was used to collect powder X-ray diffraction (XRD) patterns. An integrated multi-mode EIGER2 detector with Ni filter and CuK_α_ (λ = 1.5406 Å) operated at 40 kV, 30 mA was used. XRD profiles were recorded at a scan speed of 0.3° s^−1^ with a step size 0.02°. The 2θ range was 10–90°. The mass loss profiles of the catalysts were recorded using T.A. Instruments SDT Q600 thermogravimetric analyzer equipped with internal air cooling unit and horizontal sample holder. The measurement was carried out in N_2_ ambience with 100 mL min^−1^ flow rate. Typically, 5 ± 0.4 mg of the catalyst sample was taken in an alumina cup, and heated up to 850 °C at a heating rate of 5 °C min^−1^. An empty sample cup was loaded in the reference pan. The mass loss was continuously monitored, and the derivative mass loss was calculated to understand the regimes of major decomposition and the temperature corresponding to maximum mass loss rate.

A Micromeritics Autochem-2920 instrument was used to determine the acidity of the catalysts using ammonia temperature programmed desorption (NH_3_–TPD) method. The typical steps involved in the TPD measurement include: (a) activation of the catalyst at 300 °C in He ambience (40 mL min^−1^) for 60 min, (b) decrease in temperature to 50 °C, (c) adsorption of ammonia gas of 10% concentration in He (30 mL min^−1^) for 30 min at 50 °C, (d) evacuation of the physisorbed ammonia by flushing with He (30 mL min^−1^) for 60 min at 100 °C, and (e) monitoring the release of chemisorbed ammonia by increasing the temperature to 750 °C at 10 °C min^−1^ in continuous He flow (40 mL min^−1^). The concentration of the desorbed ammonia was measured using a thermal conductivity detector (TCD).

A Thermofisher Scientific NICOLET iS50 FTIR spectrometer was used to conduct pyridine diffuse reflectance infrared Fourier transform (DRIFT) study to assess the type of acid sites in the catalyst. The catalyst sample was taken in a high vacuum cell provided by Harrick Scientific Products, and assembled with the spectrometer. The collection of a DRIFT spectrum involved the following steps: (a) initial activation of the sample in N_2_ ambience (20 mL min^−1^) at 300 °C followed by cooling it to 100 °C, (b) recording a baseline spectrum before pyridine adsorption at 100 °C, (c) injection of 30 µL pyridine while cooling down the sample cell from 100 to 50 °C, (d) evacuating the physisorbed pyridine by increasing the temperature to 100 °C and maintaining it for 60 min, and (e) raising the temperature further to 240 °C, and recording the DRIFT spectrum after 15 min at 240 °C. The final pyridine adsorption spectra was obtained by subtracting the baseline spectrum obtained at 100 °C from that obtained at 240 °C.

High-resolution scanning electron microscopy (HRSEM) images of the catalysts were obtained using Hitachi S-4800 HRSEM, which was operated in the voltage range of 0.5–30 kV and current of 10 µA. A JEM-2100 Plus instrument (JEOL, Japan) operated at 200 kV was used to record high-resolution transmission electron microscopy (HRTEM) images of the catalysts. Typically, ∼1 mg of sample was finely dispersed in isopropyl alcohol of 30 mL using ultrasonication. This highly dispersed sample was drop casted on a carbon-coated copper grid. Finally, isopropyl alcohol was dried at room temperature for 48 h. X-ray photoelectron spectroscopy (XPS) measurements were performed using PHI5000 Version Probe III (ULVAC-PHI). X-ray core level spectra were recorded using Al Kα radiation (hʋ = 1486.6 eV). The carbon 1s peak at 284.5 eV was taken as the reference to determine the binding energy values of various elements in the catalyst.

## Ethics Statements

Not applicable.

## CRediT Author Statement

**Lakshmiprasad Gurrala:** Conceptualization, Methodology, Validation, Formal analysis, Investigation, Visualization, Writing – original draft, Writing – review & editing; **M. Midhun Kumar:** Methodology, Validation, Formal analysis; **Changyub Paek:** Methodology, Visualization, Funding; **R. Vinu:** Conceptualization, Methodology, Formal analysis, Visualization, Writing – original draft, Writing – review & editing, Resources, Supervision, Funding acquisition.

## Declaration of Competing Interest

The authors declare that they have no known competing financial interests or personal relationships that could have appeared to influence the work reported in this paper.
